# Scrofula

**Published:** 1859-11

**Authors:** 


### HALL'S JOURNAL OF HEALTH.

OUR LEGITIMATE SCOPE IS ALMOST BOUNDLESS: FOR WHATEVER BEGETS PLEASURABLE AND HARMLESS FEELINGS, PROMOTES HEALTH; AND WHATEVER INDUCES DISAGREEABLE SENSATIONS, ENGENDERS DISEASE.

*We aim to show how Disease may be avoided, and that it is best, when sickness comes, to take no Medicine without consulting an educated Physician.*

VOL. VI.] NOVEMBER, 1859. [No. 11.

### SCROFULA.

THIS is a term which takes its name from a Latin word which signifies sow, while the Greeks used a word of the same meaning, for the same disease, possibly because both Greeks and Romans found that those families suffered most with scrofula who lived after the brutish manner of swine, although the elevated and refined are not exempt from the taint. Scrofula is an error of nutrition, and hence may attack all colors, constitutions, and temperaments, but those who have light hair and fair skin are most subject to it.

All through the body, there are little bunches of vessels called "glands," which, in their natural state, are not seen, but if diseased, they swell, and when near the surface of the skin, form protuberances of irregular shapes, always rounded; hence, the ancients, who often named things from an apparent quality, called them glands, from their resemblance to an acorn or a bunch of them.

Our aliment, that which nourishes the body, must pass through what is called the absorbent glands before it is fit for nutrition; in these glands it undergoes considerable changes, but if they are in an unnatural condition, are hardened or swoolen, the changes made are not perfect—are not healthful; hence it is that scrofula is essentially an error of nutrition; the food does not give all its strength, the person may eat a great deal, and may even look stout and robust, but the appearance is deceptive—there is no endurance.

Scrofula then is manifested in an abnormal condition of the glands, giving a name to the disease according to the locality of the part affected: if in the sides of the neck, it is called king's evil, because in earlier ages, the touch of a king was thought a cure; if the glands of the joints are affected, it is white swelling; if in the lungs, it is consumption; if in the bowels, it is *tabes mesenterica*, or consumption of the bowels; the person wears away to skin and bone, and is literally consumed to death, without any cough whatever.

Why the glands of one part should become more particularly diseased than elsewhere, is simply because that part has been weakened by some violence offered to it.

But how do the glands become diseased at all, or what is the cause of their unnatural condition? In other words, what causes scrofula?

Most generally, persons are born scrofulous, in consequence of one or both parents being diseased in some way or other; but scrofula may be originated in any constitution by protracted wrong living, such as a want of personal cleanliness, or a continued dwelling in low or damp, or filthy localities, or in habitual excesses as to the animal appetites and passions.

Scrofula, like insanity or family resemblances, may pass over a generation. A man may be scrofulous, his children may not have a trace of it, yet his grandchildren may be decidedly so.

A person may be very slightly scrofulous—only a mere trace of it—so little of it as to be scarcely perceptible, or it may be of such an aggravated character as to distort the whole body.

As a general rule, scrofula shows itself in some kind of breaking out on the skin, or in some affection of the eyes. As scrofula is essentially an error of nutrition, by which the food does not impart its full strength to the system, it is characterized by a want of endurance, by a lack of power of resistance, of warding off disease, or averting "colds;" hence, scrofulous persons take cold very easily, and often describe themselves as "always taking cold," or "the least thing in the world gives me a cold," and that "cold" "settles" in the weak part; if in the tonsils, they swell internally; if the lungs are the weak part, a bad cold is the result; if in the head—and a great many have a weakness there—it is described as a cold in the head. Under certain conditions, a scrofulous person has a greater chance of long life than one who is entirely free from it, especially if well-informed and well to do, because, being conscious of a want of robustness of constitution, common sense dictates carefulness, and a systematic avoidance of those causes of ailment which observation indicates as the uniform precursors of particular symptoms, while the fact of being "well to do," gives the means of nursing, and of guarding against those exposures, over exertions and deprivations, which are the fruitful sources of sickness to the unfortunate poor.

A person born scrofulous, or becoming so after birth, need not necessarily remain so to any specially hurtful extent. If, for example, a man suffers from white swelling, or a long and tedious "running" in the neck from king's evil, the "ill humors" of the system, as they are called, seem to find vent there, leaving the constitution, comparatively, healthy, and a long life of reasonable health is the result.

Scrofula may be almost entirely "worked" out of the system in another way, as by a great and protracted change in the habits of life—such a change as involves large out-door activities for the greater part of every twenty-four hours. The same thing may be accomplished, to a great extent, in-doors, as where a sedentary life is followed by spending a large portion of each day in active employment on foot, especially if the mind is deeply and pleasurably interested in that employment; more decided results will follow, if the aid is given, meanwhile, of judicious personal habits, such as scrupulous cleanliness of body and clothing, of regular, full, and sufficient sleep; of plain, simple, and nutritious food, eaten at regular intervals of five or six hours, and nothing between, with that daily regularity which is essential to health under all circumstances.

A scrofulous person should eat fresh meats largely, and bread and fruits and berries of every description, using vegetables sparingly.

In short, whatever promotes high bodily health, promotes the eradication of a scrofulous taint; hence, it is the greatest wisdom on the part of those who are scrofulous, to study how and what gives to them the greatest general good health, and to live accordingly.

Scrofula manifests itself externally in some, as in lumps, or a variety of breakings out on the skin; in others, it causes some internal malady. In either case, the essential disease is the same; it is in the system—in the blood—and the attempt should be to eradicate, not to cover up.

If there is an external manifestation, external appliances can never radically cure, can never eradicate—their tendency is to suppress—to drive inwards, or elsewhere, generally, if not always, to find refuge in some more vital part, and the whole history reads, "cured, then died." Hence, external manifestations of scrofula are not, indeed, signs of health, but they are signs of safety. It is when measles "strike in" that there is danger.

Salt rheum is scrofula, and afflicts persons for many years, then sometimes disappears for "good and all," to the great gratification of the patient. The next report is "consumption," if in grown persons; "water on the brain," if in young children.

As to taking internal remedies, one of three things is the uniform result:

First, The medicine gradually losses its power.

Second, The system is benefited only while it is taken; or,

Third, The remedy gradually poisons the system, or impairs the tone of the stomach, thus aggravating the "error of nutrition" and hastening a fatal result.

It is greatly to be regretted that these things are not generally known; an incalculable amount of human suffering would thereby be prevented, and the unfortunate poor saved many a hard-earned dollar.

The most that can be expected as to the cure of scrofula is, that it may be kept in abeyance—may be kept under by wise habits of life, such as regularity, cleanliness, temperance in all things, and daily industry in the open air, living, the meanwhile, on plain, simple, nutritious food, of which fresh meats, ripe fruits, coarse bread, and cold water are the main. We believe that no medicine ever eradicated scrofula, or kept it under any longer than while it is taken.

